# A high birefringence liquid crystal for lenses with large aperture

**DOI:** 10.1038/s41598-022-18530-z

**Published:** 2022-08-26

**Authors:** N. Bennis, T. Jankowski, O. Strzezysz, A. Pakuła, D. C. Zografopoulos, P. Perkowski, J. M. Sánchez-Pena, J. M. López-Higuera, J. F. Algorri

**Affiliations:** 1 Institute of Applied Physics, Military University of Technology, Kaliskiego 2, 00-908 Warsaw, Poland; 2 Faculty of Mechatronics, Warsaw University of Technology, Św. Andrzeja Boboli 8, 02-525 Warsaw, Poland; 3 Institute of Chemistry, Military University of Technology, Kaliskiego 2, 00-908 Warsaw, Poland; 4 Consiglio Nazionale delle Ricerche, Istituto per la Microelettronica e Microsistemi (CNR-IMM), 00133 Rome, Italy; 5 Department of Electronic Technology, Carlos III University, 28911 Madrid, Spain; 6 Photonics Engineering Group, Universidad de Cantabria, 39005 Santander, Spain; 7 CIBER de Bioingeniera, Biomateriales y Nanomedicina, Instituto de Salud Carlos III, 28029 Madrid, Spain; 8 Instituto de Investigación Sanitaria Valdecilla (IDIVAL), 39011 Santander, Spain

**Keywords:** Adaptive optics, Liquid crystals

## Abstract

This work presents the application of an experimental nematic liquid crystal (LC) mixture (1929) in a large aperture lens. The LC material is composed of terphenyl and biphenyl derivatives compounds with an isothiocyanate terminal group and fluorinated lateral substituents. The substitution with a strongly polar isothiocyanate group and an aromatic rigid core provides $\pi$-electron coupling, providing high birefringence ($\Delta n = 0.3375$ at 636 nm and 23 °C) and low viscosity ($\eta$ = 17.03 mPa s). In addition, it also shows high values of birefringence at near infrared (0.318 at 1550 nm). The synthesis process is simple when comparing materials with high melting temperatures. The excellent properties of this LC mixture are demonstrated in a large aperture LC-tunable lens based on a transmission electrode structure. Thanks to the particular characteristics of this mixture, the optical power is high. The high birefringence makes this LC of specific interest for lenses and optical phase modulators and devices, both in the visible and infrared regions.

## Introduction

Liquid crystals (LCs) with low/medium birefringence ($\Delta$n = 0.09/0.12) are still used in display applications^1–3^. Their birefringence is sufficient for large TV screens and computer monitors, in smaller flat panels of mobile phones, automotive devices and projectors^4^. Thanks to the generation of new LC mixtures and the reduction in the pixel thickness (from 5 to 3 µm), the response times have been reduced to a few milliseconds. Despite this, current LC displays require switching times lower than milliseconds, so highly birefringent LCs ($\Delta n>0.3$) are required. Other applications requiring this property are lower frequencies of the electromagnetic spectrum (from infrared down to THz and GHz), as the optical phase shift produced by the LC is directly proportional to its birefringence and the working frequency. For example, they have been proposed as active medium in metamaterials and metasurfaces^5,6^, THz^7–9^, GHz^10,11^ and infrared devices^12^.

In the last decade, the study of common LCs in these spectral ranges has raised attention, e.g. E7, BL037, MDA-98-1602, LCMS-107, GT3-23001 and 1825^13^. In addition, several novels, highly birefringent mixtures, have been proposed. To achieve this, linearly conjugated molecules are the preferred candidates. The conjugation length can be extended by multiple bonds or unsaturated rings in the rigid core^14,15^. A comprehensive review can be found in^4^, where highly birefringent LCs with positive dielectric anisotropy are reviewed. The mesogenic properties and physical-chemical properties (viscosity, birefringence, refractive indices, dielectric anisotropy and elastic constants) of compounds that are cyano, fluoro, and isothiocyanato derivatives of biphenyl, terphenyl, quaterphenyl, tolane, phenyl tolane, phenyl ethynyl tolane, and biphenyl tolane are compared, with a birefringence ranging between 0.2–0.5^4^.

The field is still very active and, more recently, several works have been reported. For example, in^16^, the authors synthesized 20 LC mixtures, both symmetrical and non-symmetrical bistolanes with terminal alkyl, alkoxy and alkylsulfanyl chain and lateral methyl or ethyl group. The compounds with a nematic phase in a broad temperature range showed a high birefringence value (> 0.4). Similar values have been also shown based on thieno[3,2-b]thiophene-based compounds^17^, thieno[3,2-b]thiophene with a – C $\equiv$ C – triple bond (0.40–0.48)^18^, and benzoxazole-terminated mesogenic compounds with fluoro substituent at different positions (0.45)^19^. Higher values (0.66) have also been demonstrated in LC compounds with isothiocyanate and naphthyl groups. The melting points and enthalpy values of these LC compounds were higher than those of corresponding compounds with the phenyl group^20^. Finally, ultrahigh birefringence has been demonstrated in some isothiocyanato biphenylbistolane compounds (0.7–0.8)^14^.

One of the main problems of these ultrahigh-$\Delta n$ mixtures is their high viscosity, which increases the response time and dispersion in the visible range. Another problem can be the UV stability due to the long absorption tail^14^. For this reason, some applications that require fast switching times and better optical qualities are limited to lower $\Delta n$ values (0.3–0.4)^21^, e.g. in optical communications^22^, or adaptive LC-lenses^23^. In the first work, two high-birefringence and low-viscosity nematic mixtures (LCM-1107 and LCM-2018) are demonstrated to work in phase-only LCoS panels intended for 6G communications; the birefringence is 0.312 for LCM-1107 and 0.344 for LCM-2018. The second work proposes a novel LC mixture composed of three different rod-like LCs. Structures belong to fluorine substituted alkyl-alkyl phenyl-tolanes, alkyl-alkyl bistolanes and fluorine substituted 4-[(4-cyanophenoxy)carbonyl]phenyl 4-alkylbenzoates. This material possesses high birefringence ($\Delta$n = 0.32) as well as high dielectric anisotropy ($\Delta \varepsilon$ = 6.3), with the unique property of frequency-controlled phase modulation, as in a dual-frequency liquid crystal, with the difference that its dielectric anisotropy goes to zero instead of being negative at high frequencies^24^.

The field of LC-tunable lenses is very active at the moment^25,26^, as they can be used in ophthalmic applications^27–29^, mobile phones^30^, autostereoscopic devices^31,32^, plenoptic capture systems^33,34^, virtual reality displays^35^, to name some key enabled applications. In recent years, numerous structures have been proposed, e.g., by using curved electrodes^36,37^, built-in dielectric layers^38–40^, multielectrodes^41–44^ and modal lenses^27,30,45,46^. Some disadvantages of curved electrodes and dielectric layers are the high required voltages due to the distance of the electrode to the LC layer. This effect is avoided with the multielectrode technique that usually is placed in contact with the LC. In addition, the phase profile can be precisely controlled thanks to the possibility of applying different voltages. Despite this, the voltage control is usually complex as several voltage sources are required, consequently the fan-in (number of input signals) is large. Modal lenses use a high resistivity layer that distributes the voltage across the active area to solve this issue. A hyperbolic voltage profile can be obtained using only one or two voltage sources. However, the use of very thin layers (to achieve high resistivity) generates several issues, e.g., complicated fabrication (due to uniformity and part-to-part variability), environmental instability and temperature sensitivity^47,48^.

The transmission-electrode technique solves all these issues, as it consists of an ITO electrode with a high aspect ratio (length over width). The produced resistance is high, maintaining the current low but distributing the voltage similarly to modal lenses. Thanks to avoiding high-resistivity layers, the associated drawbacks are missing. This technique has been demonstrated in various types of LC lenses, e.g., axicons^47,49,50^, Powell^51^ and aspherical^48,52,53^. In^48^, the transmission electrode has a spiral configuration and it has only one contact, so phase changes are performed through frequency sweeping. One of the main challenges of LC lenses is to produce high optical power combined with a large aperture. As already commented, there are many highly birefringent LC mixtures, some of them with a birefringence reaching the extreme value of 0.8. However, for LC lenses, there is a clear need for novel mixtures with more moderate birefringence but better optical quality and response time. For this reason, in this work, a high-birefringence and low-viscosity nematic mixture (1929) that was chemically presented previously in^54^, is demonstrated to work in a large aperture LC lens based on the novel transmission electrode technique^52^. As commented before, the experimental LC mixture has a high birefringence, $\Delta n = 0.3167$ at 20 °C and low viscosity $\eta$ = 17.03 mPa s^54^. A dispersion analysis is made in the present work revealing that this LC can also be used in near infrared applications ($\Delta n = 0.3375$ at 636 nm and $\Delta n = 0.318$ at 1550 nm, 23 °C). The fabrication process is simple when compared with materials with high melting temperatures. In addition, the lens optical quality is demonstrated through phase profiles, point spread functions and refocusing images.

## Liquid crystal mixture and structure

**Figure 1:**
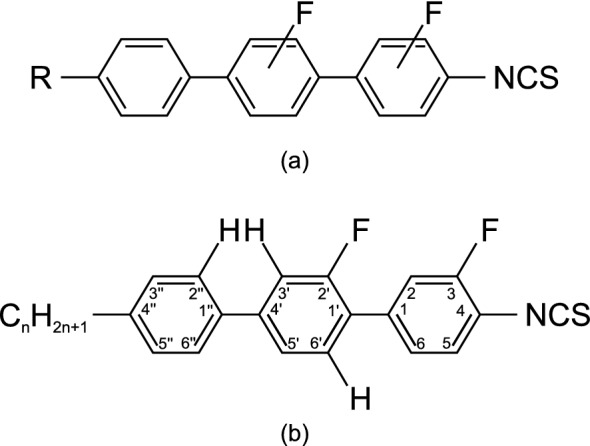
(**a**) General formula of mixture 1929. (**b**) The chemical formula of prepared isothiocyanatoterphenyls^54^.

The nematic liquid crystalline material used in this study is composed of terphenyl and biphenyl derivative compounds with an isothiocyanate terminal group and fluorinated lateral substituents, shown in Fig. 1. The general formula of the liquid crystal compounds indicates that the fluorine atoms may be substituted at any position on the benzene rings. The substitution with a strongly polar isothiocyanate group together with an aromatic rigid core provides $\pi$-electron coupling, thus increasing the birefringence of the material: $\Delta n=0.364$ at 636 nm and 23 °C. The presence of fluorine atoms lowers the melting and clearing temperature, simplifying the preparation process compared with materials with high melting temperatures. The phase transition temperature from nematic to isotropic phase is 96.2 °C. Specifically, the mixture was calculated from the CSL equation^55^ as an eutectic composition comprising ethyl, butyl, pentyl members of compound Fig. 1b and 4′-propyl- 3-fluoro-4-isothiocyanatobiphenyl. The compound of Fig. 1b has alkyl chains containing two, three, four and five carbon atoms. They range from n = 2 to n = 5. Some members belonging to four families among these homologues series were prepared earlier (see refs.^56,57^). The positions of fluorine atoms were mainly chosen in such a way as to limit the twisting of the neighbouring benzene rings, which are responsible for the decrease of the $\pi$-electron conjugation. The mixture was prepared by weighing out an appropriate quantity of the individual components and then heating above the transition temperature to an isotropic liquid and stirring. Next, silica gel was added to the cooled mixture, mixed and after 24 hours, filtered through a vacuum system.

**Figure 2:**
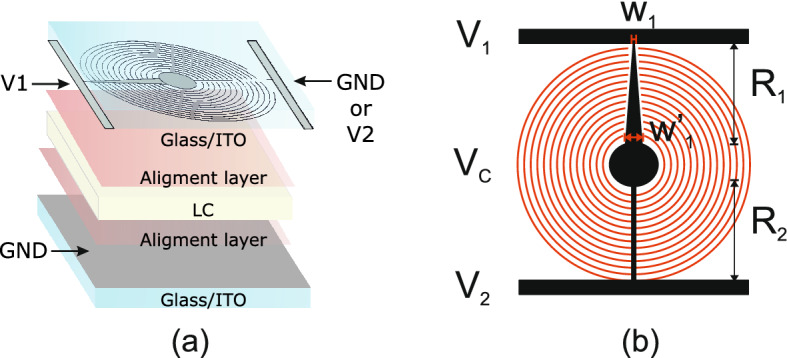
(**a**) Schematic depiction of the LC-tunable large-aperture lens and its various constituent parts. The top substrate shows the electrode configuration for the distribution of the applied voltage profile. (**b**) Detail of the top substrate electrode. The figure was generated using Inkscape software with version no. 1 and link https://inkscape.org/es/.

Two types of cells were filled with this LC mixture. The first one is a single-pixel cell used to measure the electro-optical properties of the LC. In this case, a continuous electrode in both substrates, having an active area of 1 cm$^{2}$ was used. The thickness of the measured cell was 10 µm. The second one is intended to demonstrate its use for LC lenses, as shown in Fig. 2a. The transmission electrode technique is chosen as the enabling technology^52^. This technique is easy to fabricate, as a basic sandwiched LC structure is used. As can be observed in Fig. 2b, the top-substrate contact consists of a transmission electrode that generates a non-linear voltage profile (from $W_1$ to $W_1'$). By applying a voltage at $V_1$ and $V_2$, the resulting voltage divider formed by $R_1$ and $R_2$ produces a controlled voltage at $V_C$. This voltage is distributed across the active area through densely packed concentric electrodes (red lines). The active area diameter is 1 cm and the gap between the adjacent concentric electrodes is 10 µm. One ITO-coated substrate (with surface resistance $R_\text {s}=100$ $\Omega$/sq) is photolithography etched to produce the electrode configuration using a photomask. For planar alignment, the substrates were coated with polyimide alignment layer SE-130 (from Nissan Chemical Industries, Ltd.). Then, they are mechanically rubbed to define the alignment direction for the LC molecules on the surface. Spacers of 80 µm diameter, mixed with optical glue, were deposited to separate the upper and bottom substrates and fix the thickness of the active LC cell. Finally, the investigated experimental LC mixture 1929 infiltrated the cavity.

## Setup and experimental results

One important LC parameters are their dielectric constants that determines the threshold voltage of the LC. This parameter is very dependent on frequency and temperature and for this reason, a detailed study is shown in this section. In addition, another essential characteristic of LCs is their optical anisotropy or birefringence. This feature, which can be dynamically tuned by applying an external low-frequency AC voltage, was characterized by measuring the refractive index for two different voltages. As it happens with dielectric constants this parameter varies with the frequency or wavelength (dispersion). The birefringence determines essential characteristics such as the difference in the phase modulation when different wavelengths are considered, and it is presented in this section. Also a thermal stability study is shown, to demonstrate a good response to temperature variations. Finally, as a high birefringence value is demonstrated, as a case study a large aperture LC lens using the proposed LC material is presented. The results show an optical power of more than $\pm 1$ Diopter for a 1 cm aperture lens.

### UV–VIS–NIR spectra

**Figure 3:**
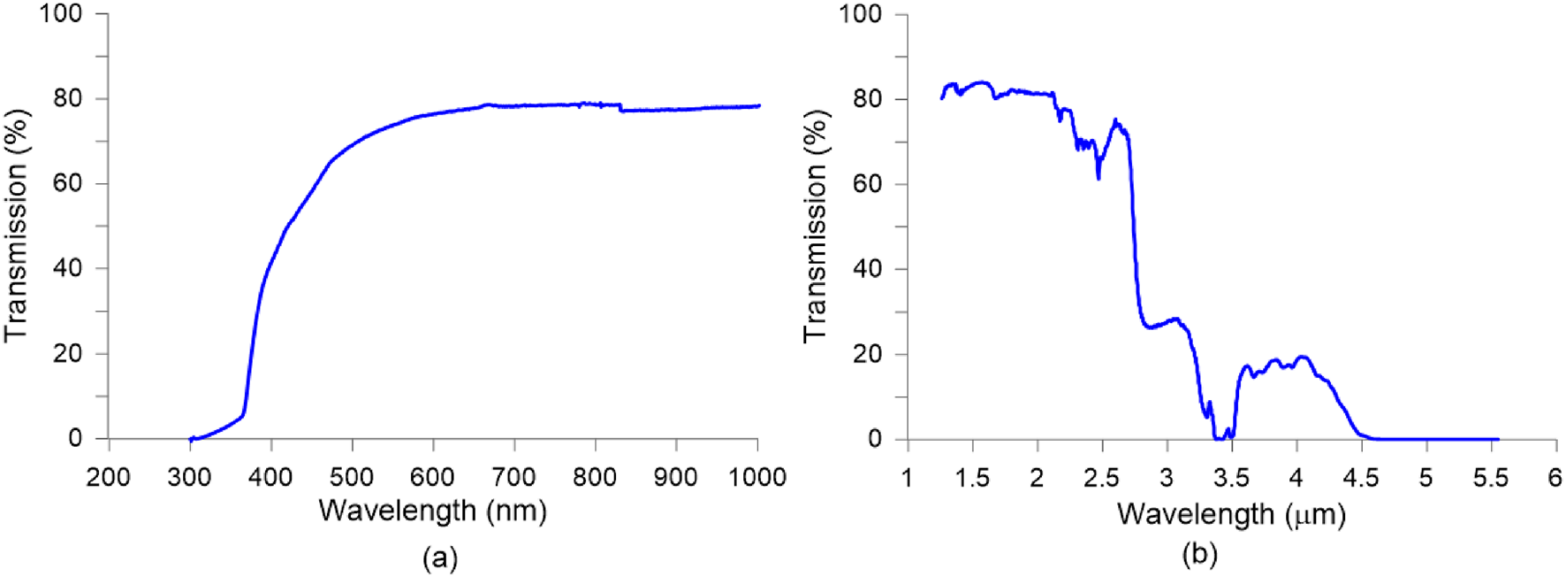
Transmission spectra for: (**a**) UV–VIS and (**b**) NIR.

The transmission of the 80 µm sample is measured by using two different spectrometers. It has to be noted that all layers (glass, ITO, alignment and LC) are included. To measure the spectra in the ultraviolet (UV) and visible (VIS) ranges a UV-3600 SHIMADZU spectrophotometer (SHIMADZU, Japan) is used. It is based on a high-performance, grating-grating double monochromator, which achieves a low stray-light level with high resolution. The UV-3600 provides precise transmittance or reflectance measurements in the ultraviolet to near-infrared regions. Conventional spectrophotometers use a PMT (photomultiplier tube) for the ultraviolet and visible region and a PbS detector for the near-infrared region. Neither detector, however, is very sensitive near the wavelength of 900 nm, preventing high-sensitivity measurements in this range. The UV-3600 makes it possible to take high-sensitivity measurements in the switchover range by incorporating an InGaAs detector. Switching between the PMT and the InGaAs detector is possible in the range of 700 to 1000 nm (the default switchover wavelength is 830 nm). For a spectra from 1 to 5.5 µm, a Fourier transform infrared (FT-IR) spectrophotometer is used (NICOLETiS10 from Thermo Scientific). This device includes diamond-turned mirrors and locked-in-place optical elements to offer excellent wavelength accuracy without requiring software for spectral correction. The automatic atmospheric interference suppression removes water and carbon dioxide from the spectra, without the need to select a reference spectrum. In addition, the dynamic alignment offers superior sweep speeds and performance. The results of Fig. 3 shown a good performance of the mixture 1929 in the NIR (until 2.5 µm) validating its use in this spectra range.

### Birefringence characterization

**Figure 4:**
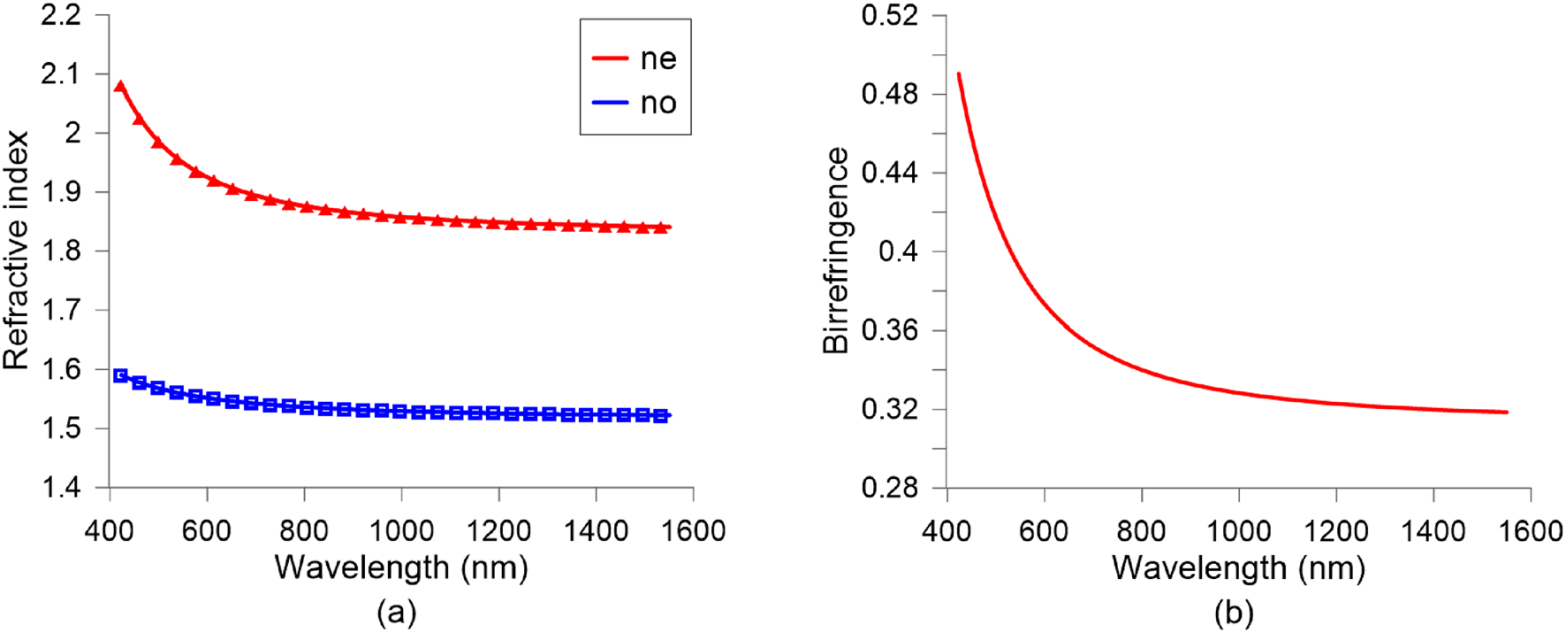
Wavelength dispersion of: (**a**) the extraordinary ($n_\text {e}$) and ordinary $n_\text {o}$ LC indices (symbols are experimental data and solid line the Cauchy fit) and (**b**) the LC birefringence ($\Delta n = n_\text {e}-n_\text {o}$).

The setup is based on a prism coupler (Metricon, model 2010). The Metricon Model 2010 Prism Coupler utilizes advanced optical waveguiding techniques to rapidly and accurately measure both the thickness and the refractive index/birefringence of dielectric and polymer films as well as the refractive index of bulk materials. This device offers unique advantages over conventional refractometers and instruments based on ellipsometry or spectrophotometry, such as high accuracy ($\pm 0.0005$), rapid characterization (20 s) and wide index measurement range (1.0–3.35). Coupling profiles as a function of incident angle were analyzed with the 2010 system software. For the measured extraordinary and ordinary LC refractive indices are plotted in Fig. 4a, where the symbols are the experimental data and the solid line is the Cauchy fit. Based on the previous data the birefringence can be calculated as $\Delta n = n_\text {e}-n_\text {o}$, Fig. 4b.

The characterization wavelength range spans from 400 to 1600 nm, with a birefringence variation from 0.490 to 0.318, respectively. This demonstrates a very high birefringence even for near-infrared applications. As can be seen, the higher the wavelength, the lower the birefringence. This tendency is usually captured by different models, among which one of the most accepted is the Cauchy equation. The dispersion of the LC indices is, accordingly, described by

1 $$\begin{aligned} n_\text {o,e}(\lambda ) = A + \dfrac{B}{\lambda ^{2}} + \dfrac{C}{\lambda ^{4}}, \end{aligned}$$

The Cauchy coefficients for the indices of the LC mixture1929 were obtained through fitting the measured data to Eq. (1) and they are reported in Table 1. These parameters are instrumental in modelling the LC behaviour in different simulations.

**Table 1:** Cauchy coefficients for the indices of the LC mixture 1929 ($\lambda$ in nm)

	*A*	$B (10^{4})$	$C (10^{8})$
$n_\text {o}$	1.517	1.2360	1.069
$n_\text {e}$	1.83	2.331	38.560

### Relative permittivity characterization

**Figure 5:**
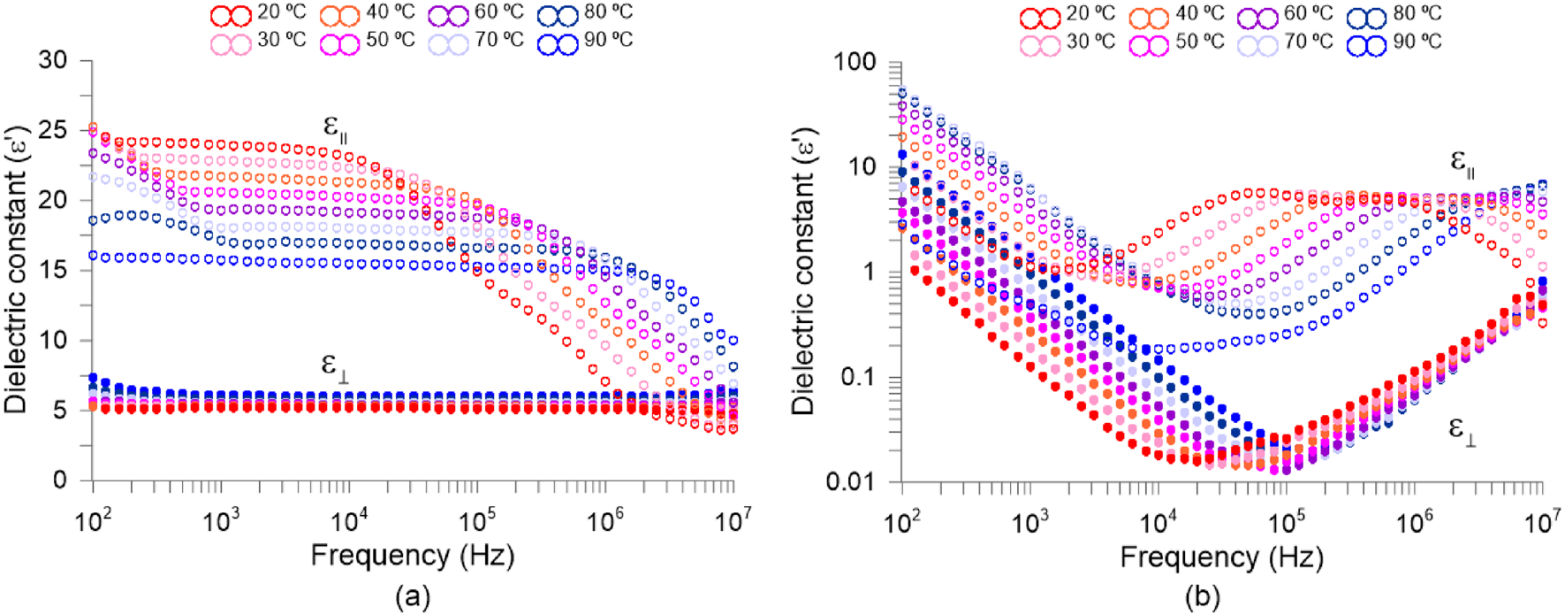
Frequency dependence of complex relative permittivity. (**a**) Real ($\varepsilon '$) and (**b**) imaginary $\varepsilon ''$. indices.

**Figure 6:**
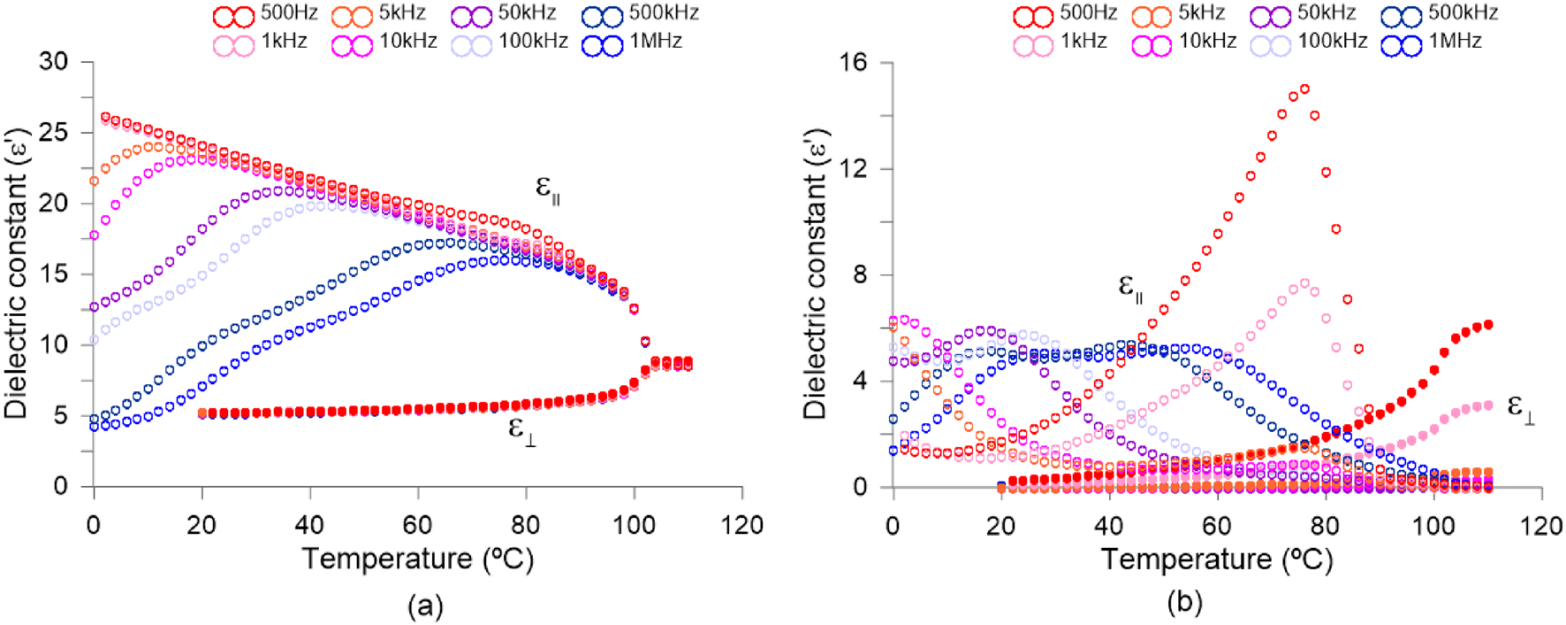
Temperature dependence of complex relative permittivity. (**a**) Real ($\varepsilon '$) and (**b**) imaginary $\varepsilon ''$. indices.

The parameter used to determine the LC interaction with electrical signals is often expressed by permittivity, $\varepsilon$, (related in a quadratic proportion to refractive index). The absolute permittivity means the resistance encountered by an electrical field in a determined medium, that is, the material ability to transmit an electrical field. The relative permittivity is the permittivity expressed as a ratio relative to the permittivity of vacuum. This parameter is commonly known as dielectric constant and is also affected by the elongated shape of the LC molecules. The dielectric constant is a complex number with the imaginary part associated with dielectric losses and the real part with the degree to which a material can be polarized. As with birefringence, the maximum difference between dielectric constants in each molecular axis is known as dielectric anisotropy ($\Delta \varepsilon = \varepsilon _\parallel -\varepsilon _\perp$). Dielectric spectroscopy measurement was performed to confirm the electric properties of the investigated nematic mixture. We used a 3 µm thin cell with gold electrodes. Standard cell^58^ was prepared in our clean room. To get the planar alignment, polyimide SE130 was used. The cell was filled with the capillary action in isotropic phase (at temperature of around 110 °C). Measurement was performed during the cooling cycle. We used a HP 4192A impedance analyzer. The temperature was controlled using Linkam TMS 92 and heat unit THMSE 600. Gold electrodes made the measurements free of parasitic distortion^59^ for frequencies up to 5MHz. The results are displayed in two formats, complex dielectric constant vs frequency (Fig. 5) and versus temperature (Fig. 6). Planar ($\varepsilon _\perp '$) and homeotropic ($\varepsilon _\parallel '$) orientation are obtained by applying 0 and 15 DC voltage respectively. Due to the positive electric anisotropy, the liquid crystal was reoriented under the DC field. Ions are not important as the sample was well purified.

As can be observed in Fig. 5a, for real dielectric constant two well visible relaxations are detected, around 10 and 100 kHz for 20 °C. They can be also observed in the imaginary part of the dielectric constant, Fig. 5b. These are molecular S-modes for different molecules building the investigated mixture. In Fig. 6 the complex dielectric constant versus temperature for planar ($\varepsilon _\perp '$) and homeotropic ($\varepsilon _\parallel '$) orientation is shown for several frequencies. One can see that in planar cell ($\varepsilon _\perp '$—wing) we do not see any dispersion. It means that either the molecules do not possess the dipole moments perpendicular to the molecular axis or they possess this component of dipole moment. Still, the relaxation frequency of rotation around the long molecular axis is higher than the frequency available in the experiment. In homeotropic orientation ($\varepsilon _\parallel '$— wing) one can see the strong dispersion. The slope is not uniform in Fig. 6, hence we can observe more than one relaxation in the investigated mixture. The molecules giving their contribution to electric response possess longitudinal components of dipole moments. In electric response, we see two strong S-modes (motions around short molecular axes). It is worth underlining, that investigated mixture is close to being a good candidate to obtain a dual-frequency nematic mixture^60^: $\varepsilon _\parallel '$—wing are close to cross $\varepsilon _\perp '$—wing at low temperatures (see plot 1 MHz at temperature 23 °C). One can see that dielectric anisotropy for 30$^{\circ }$C at 1 kHz is $\Delta \varepsilon$ = 17.7, while for 20 °C at 1kHz is $\Delta \varepsilon$ = 18.8. When we use higher frequencies the electric anisotropy goes down and reaches e.g. $\Delta \varepsilon$ = 4.8 for 30 °C at 1 MHz. Finally, as can be observed, the phase transition is at 104 °C

### Thermal stability

The investigation of thermal stability was carried out by differential scanning calorimeter (DSC). DSC is a thermal analysis technique that measures the heat flow of a substance in the presence of a reference material, while the substance is heated, cooled or kept at constant temperature. It allows detecting endothermic and exothermic effects, measuring reaction enthalpies, determining temperatures that characterize the various thermal transitions, as well as determining the heat capacity. It can be applied in the study of polymers, pharmaceuticals, food, etc. DSC reveals the presence of phase transition in the LC by detecting the enthalpy change associated with each phase transition. The level of enthalpy change involved at the phase transition provides information about the types of phases involved. The DSC thermograms showing the variation of heat flow (mW) with temperature (°C) in the heating and cooling cycles of the LC sample is measured by using a DSC Netsch 204 F1 Phoenix. The DSC measuring cell consists of a high-conductivity cylindrical silver block with integrated heating coil for broad thermal symmetry (3D symmetry) in the sample chamber, cooling ports for liquid nitrogen or compressed air cooling and a cooling ring for the connection of the intracooler (also with simultaneous liquid nitrogen cooling). The gas-tight construction and integrated mass flow controllers for purge and shield gases allow coupling in Fourier transform infrared or mass spectrometers for gas analysis.

**Figure 7:**
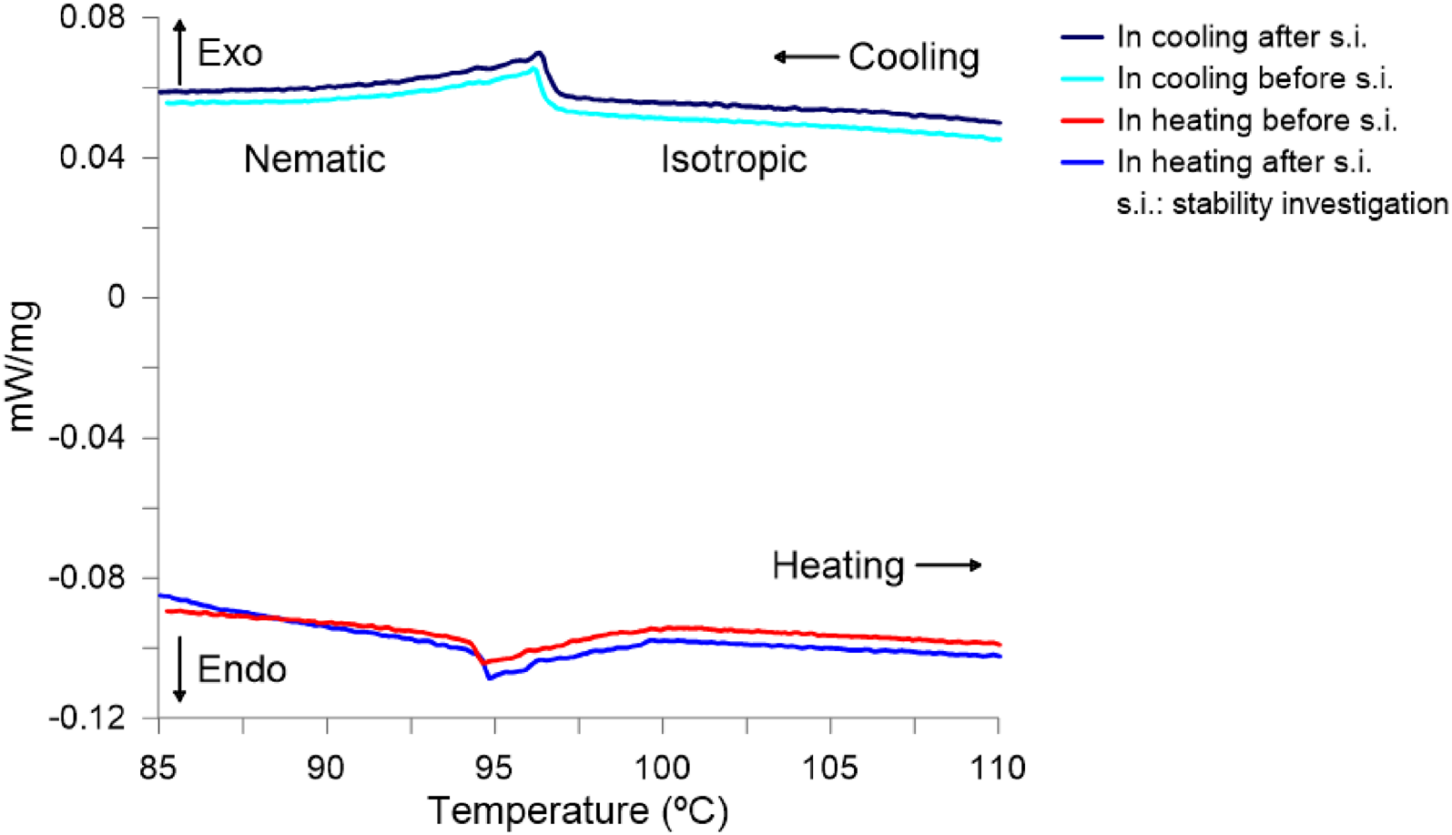
DCS thermographs upon heating/cooling cycles of mixture 1929.

The DSC measured the heat flow (mW/mg) of mixture 1929 in heating and cooling cycles (cyan and red curves in Fig. 7). We repeated the DSC measurement after the material was stored at 80$^{\circ }$C for three hours. The first cycle was increased with the rate of 5 °C/min to 110 °C and then decreased to 85°C with the same rate (blue and black curves in Fig. 7). The temperatures of the corresponding peaks upon heating and cooling differ from each other only slightly for the I-N transition. One endothermic peak along the heating indicates that the temperature of the phase transition from nematic to isotropic phase (N-I) (measured in the heating cycle) is 94.7 °C before storing for 3 h the material at 80 °C and 94.9 °C after the storage process. The temperature measured in the cooling cycle equals 96.2 °C and 96.3 °C before and after the storage test. Those results show that the temperature of the phase transition from N-I does not change after keeping the material at 80 °C for 3 h indicating good thermal stability of the material. In the case of the appearance of decomposition products, this temperature would be several degrees lower. It is important to note, that the research was conducted in the presence of a nitrogen atmosphere without oxygen.

### Case study: large-aperture LC lens

**Figure 8:**
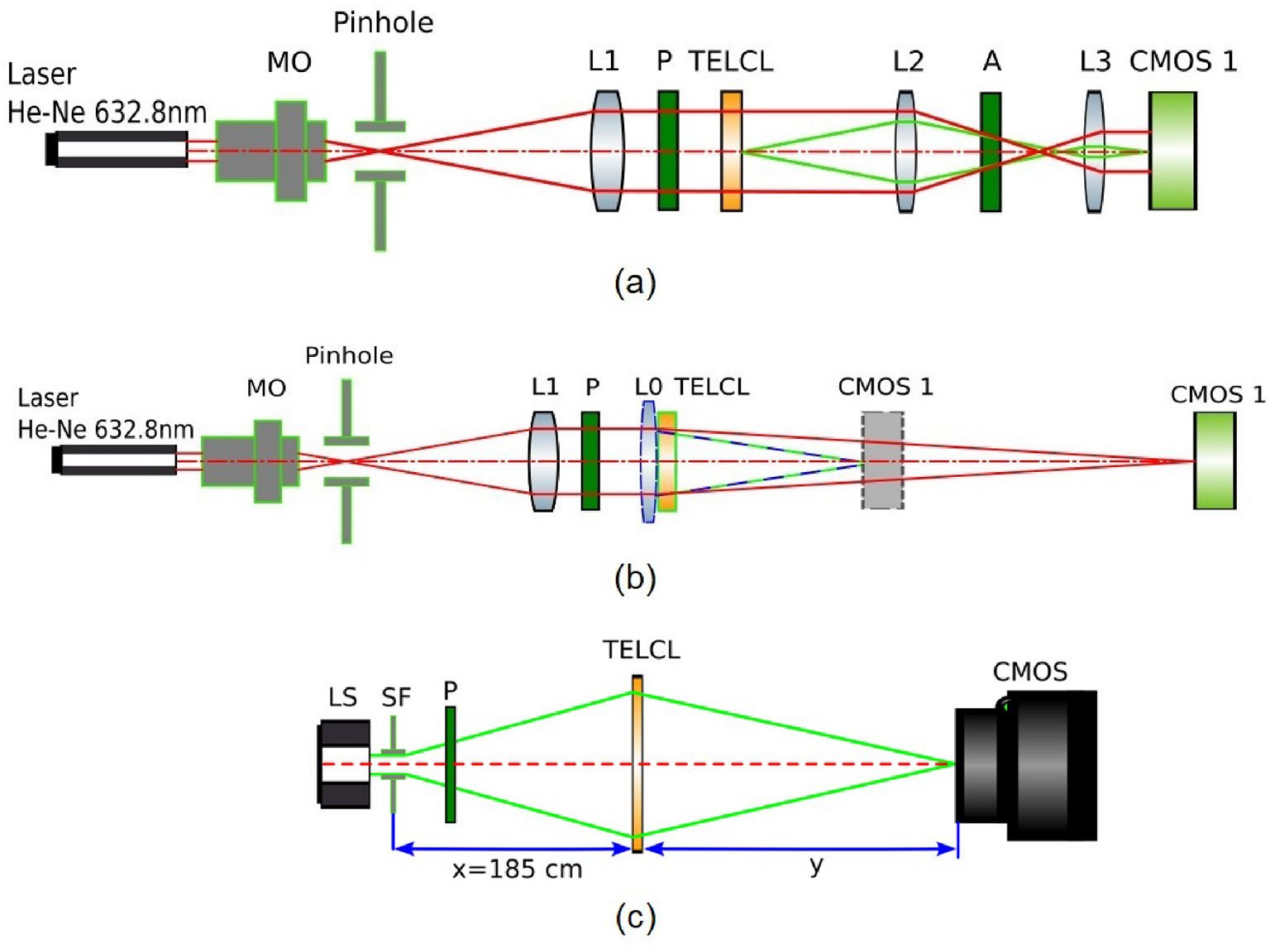
(**a**) Schematic of the optical system for measuring (**a**) the fringe patterns by placing the LC lens between crossed polarizers, (**b**) the focal length of TELCL and (**c**) the MTF function. The figure was generated using Inkscape software with version no. 1 and link https://inkscape.org/es/.

To demonstrate the LC performance in a real application, the lens described in the structure of Fig. 2 is characterized. The optical system depicted in Fig. 8a was employed to measure the fringe patterns of the transmission electrode LC lens (TELCL). From this data, the phase retardation and optical power are estimated. A collimated He-Ne laser (wavelength of 632.8 nm) is used as a light source. The TELCL is placed between crossed polarizers to measure the interference pattern between extraordinary and ordinary rays.

**Figure 9:**
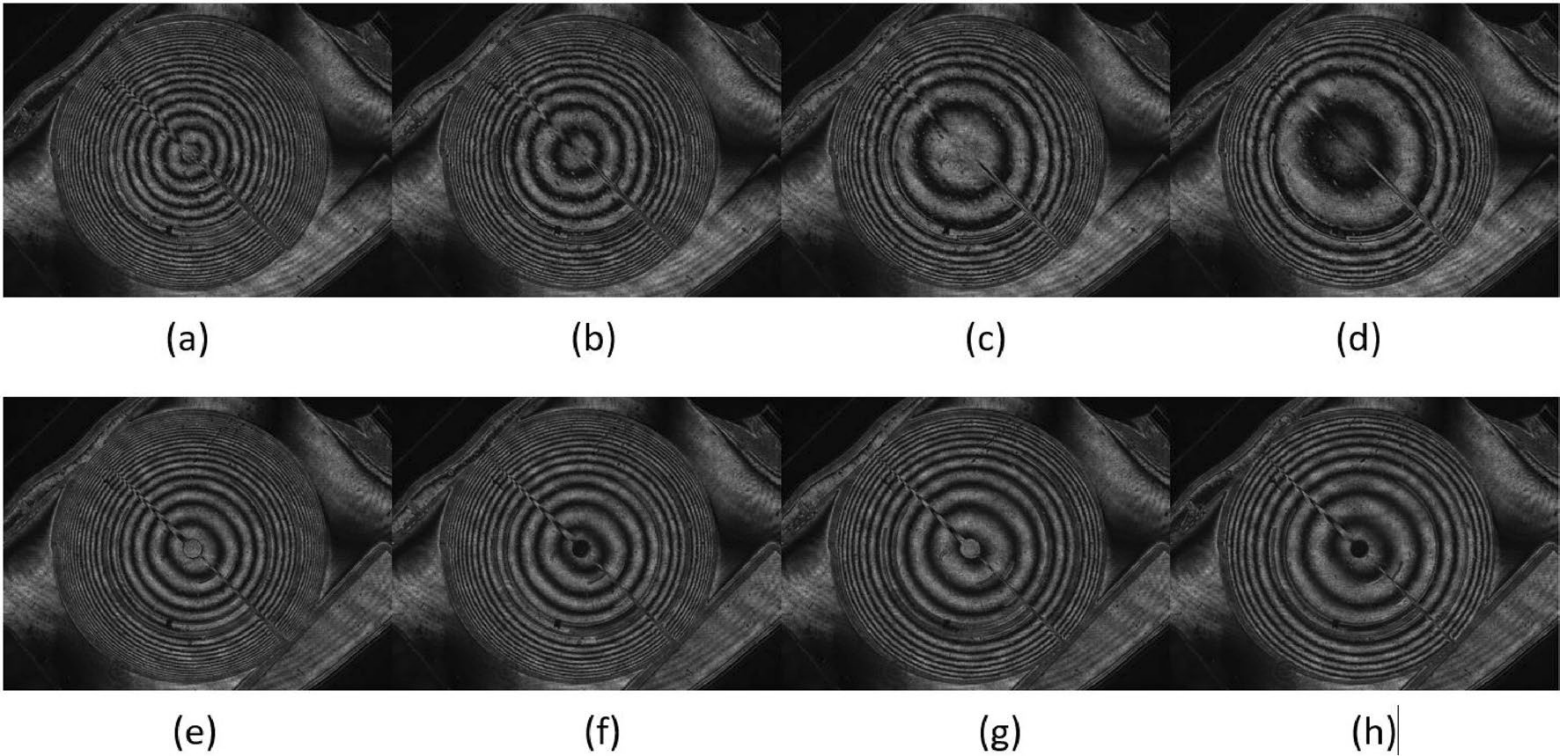
Interference patterns measured by placing the LC lens between crossed polarizers. Positive lens (V$_{\text {RMS}}$ values): (**a**) $V_1=1.75$, $V_2=0.5$, (**b**) $V_1=1.5$, $V_2=0.5$, (**c**) $V_1=1.35$, $V_2=0.5$, (**d**) $V_1=1.25$, $V_2=0.5$. Negative lens (V$_{\text {RMS}}$ values): (**e**) $V_1=1.25$, $V_2=3.5$, (**f**) $V_1=1.4$, $V_2=3.5$, (**g**) $V_1=1.55$, $V_2=3.5$, (**h**) $V_1=1.65$, $V_2=3.5$. The figure was generated using Spinview software with version no. 3.1 and link https://www.flir.es/products/spinnaker-sdk/?vertical=machine+vision &segment=iis.

The image of the interference patterns is adapted to the CMOS 1 sensor through two biconvex lenses (L2 and L3) that resize the image. Thanks to the interference patterns, the phase retardation can be estimated by measuring consecutive rings (maximum-minimum transmittance), which gives the phase profile in steps of $\pi$. Some examples of symmetric positive-negative optical powers are shown in Fig. 9. In addition, a focal length measurement was performed to confirm the optical properties of TELCL and its focusing and defocusing ability. The setup for estimating focal lengths in positive and negative modes can be found in Fig. 8b. A collimated He-Ne laser (wavelength of 632.8 nm) is used as a light source. A light beam passes through a polarizer whose polarization axis is parallel to the liquid crystal director. While measuring positive focal lengths of TELCL there is no L0 lens in the setup (light rays represented as red line). Therefore, the focal estimate is the distance from TELCL on which the tiniest light spot can be acquired on CMOS1 camera. For measuring negative focal lengths, an additional biconvex lens (L0) is added close to TELCL (light rays are represented as a blue-green line). Therefore, the distance on which the smallest spot can be seen on CMOS camera is the focal length of the two lens systems (biconvex and TELCL). Because they were near each other, we can assume that their optical powers were summed up. By knowing the focal length of the biconvex lens, the negative focal distances of TELCL were calculated. Finally, in order to calculate MTF of TELCL, the setup for imaging point source is depicted in Fig. 8c. A SLS2021 broadband light source (LS) illuminate the 40 µm spatial filter (SF) creating a spherical wavefront. A light beam passes through a polarizer (P) whose polarization axis is parallel to the liquid crystal director. The TELCL was placed at $x = 185$ cm distance from point source—the distance larger then speculated focal length of the lens. The smallest spot of maximal intensity was spotted at some distance with CMOS camera (FLIR BFS-U3-28S5M) with additional objective of 4 $\times$ magnification.

**Figure 10:**
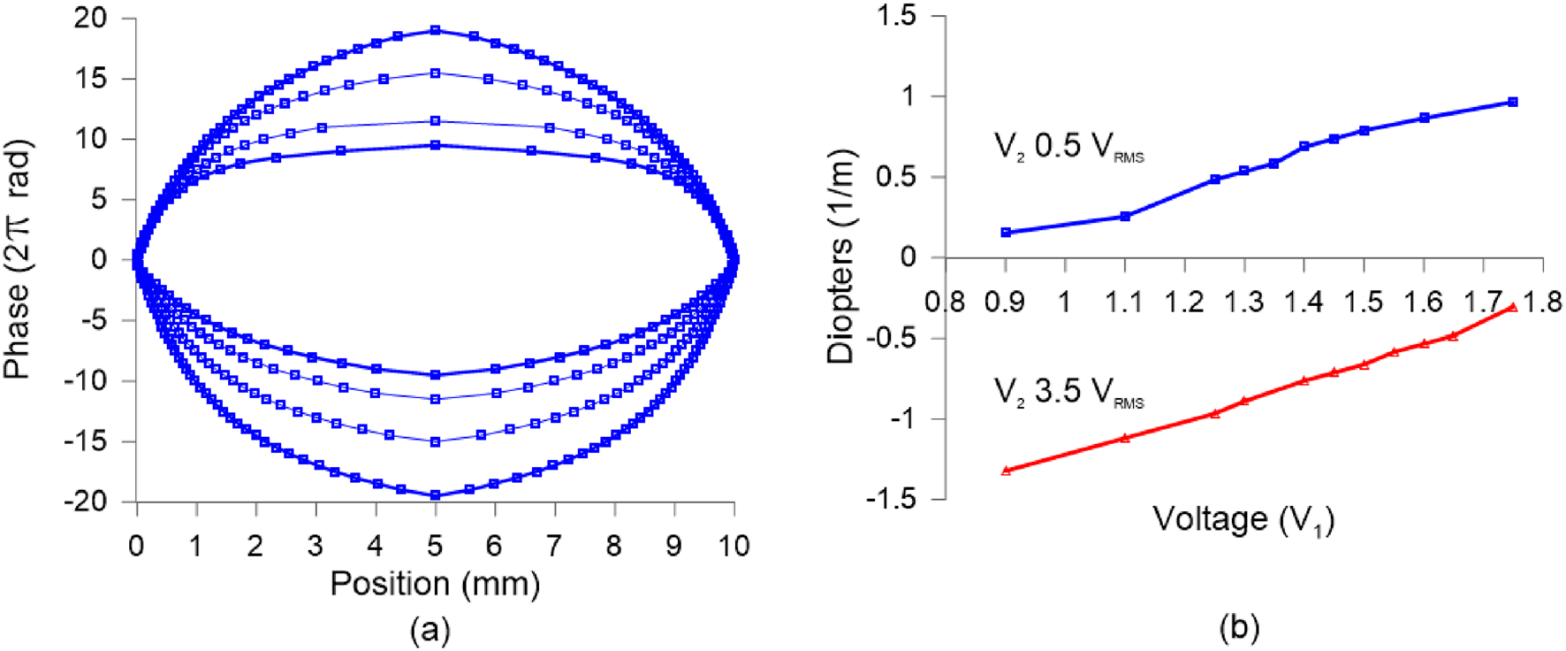
(**a**) Experimental phase shift profiles extracted from the fringe patterns of Fig. 5. (**b**) Optical power for different applied voltages.

**Figure 11:**
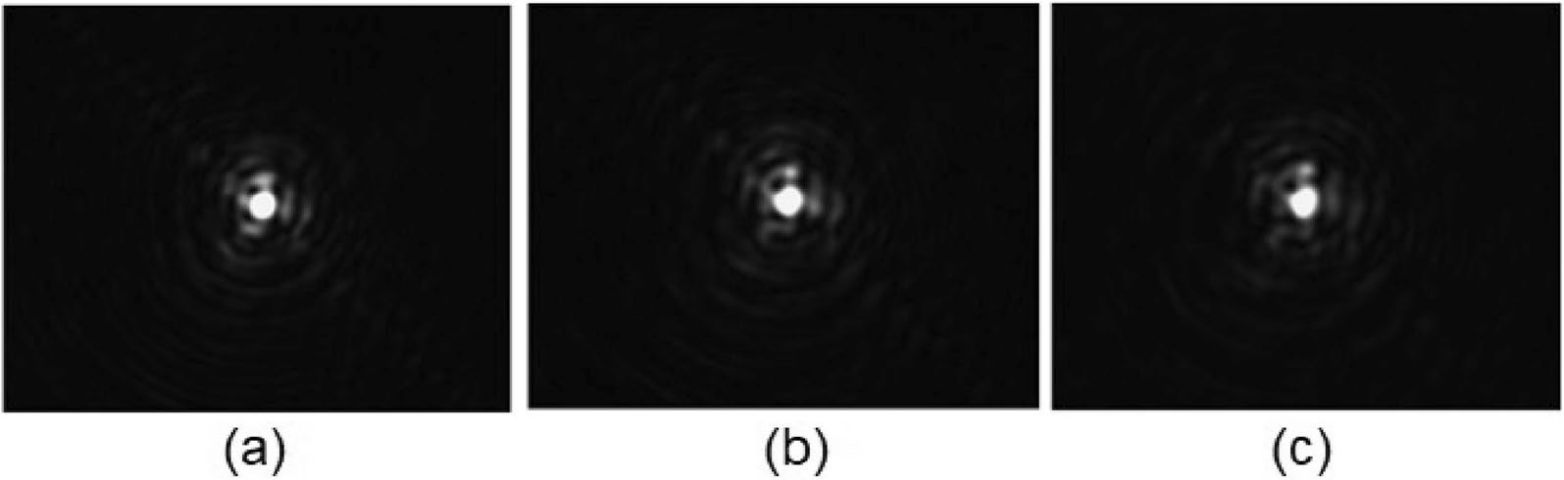
Negative lens focal spot for (**a**) $\hbox {f} = -200$ cm, (**b**) $\hbox {f} = -147$ cm, (**c**) $\hbox {f} = -120$ cm.

By considering a line crossing through the middle of the lens, the measured phase steps describe the 2D phase profiles. The profiles corresponding to the patterns of Fig. 9 are calculated by using MATLAB$^{\circledR }$ R2020a and the results are shown in Fig. 10a. As can be observed, the shapes are almost parabolic due to the voltage distribution of the variable transmission electrode. In Fig. 10b, the optical power in Dioptres reveals that the maximum optical power is almost $-1.5$ Dioptres (1/*f*) which is three times higher than a previous report using the same structure but with a standard, moderately birefringent LC^52^. In addition, Fig. 11 shows a good focal spot quality for the negative lens for 3 different optical powers.

**Figure 12:**
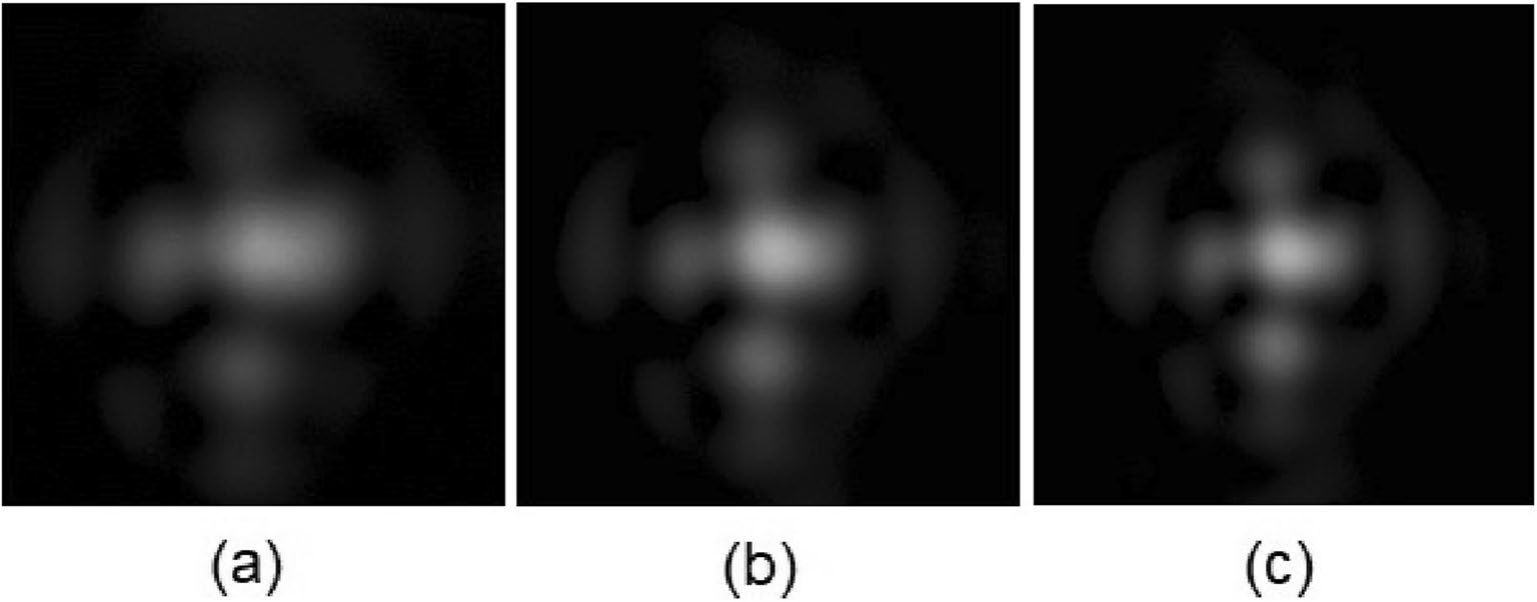
Different PSFs for (**a**) $V_1= 1.75$  V$_{\text {RMS}}$ and $V_2 = 4.5$ V$_{\text {RMS}}$. (**b**) $V_1= 1.80$ V$_{\text {RMS}}$ and $V_2 = 0.6$ V$_{\text {RMS}}$ (**c**) $V_1= 1.85$ V$_{\text {RMS}}$ and $V_2 = 0.6$ V$_{\text {RMS}}$.

It has to be noted that other lenses of the setup also affect this effect. For this reason, the true PSF taken from the setup of Fig. 8c is shown in Fig. 12. For chosen cross-section through acquired PSFs Fourier analysis was carried out. The cross-section intensity signal was convoluted with sinusoidal signals of different frequencies. The decrease in amplitude in convoluted signal corresponds to the decrease of modulation for a given frequency. The calculation was carried out for four cross-sections of every image—vertical, horizontal, and diagonal.

**Figure 13:**
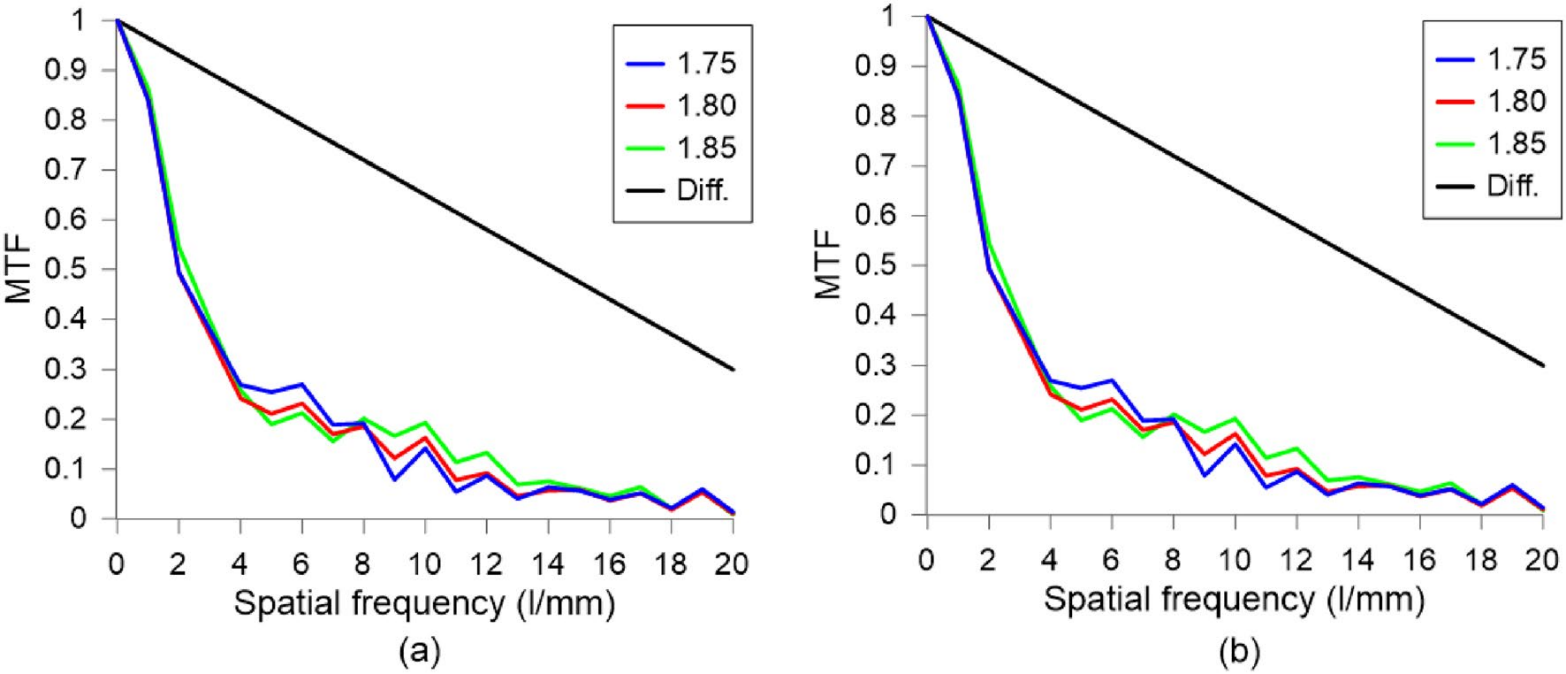
MTFs for (**a**) horizontal and (**b**) diagonal cross-sections for different applied voltages and the diffraction limit (Diff.).

The results were compared for adequate cross-sections. Additionally, the MTF curve of a perfect aberration-free lens of 1 m focal length and aperture of 10 mm was placed on the graphs. The value of focal length is similar to the ones achieved in TELCL and the diameter is the same. The diffraction-limited MTF curve was simulated in Lambda OSLO software (Diff. in Fig. 13).

The MTF curves show that for different optical powers resolution of the TELCL is similar. For higher powers, it is slightly, but not significantly better. On the other hand, there is a significant difference in MTF curves for different cross-sections. For horizontal and vertical ones, half maximum of contrast appears for 2–3 lines per mm. The cut-off-frequencies (20$\%$ of contrast) are at 5–7 lines per mm. As for diagonal cross-sections ones half maximum of contrast appears at 4–6 lines per mm and cut-off at 9–11 lines per mm. It has to be noted that MTF would be enhanced by reducing the LC thickness.

**Figure 14:**
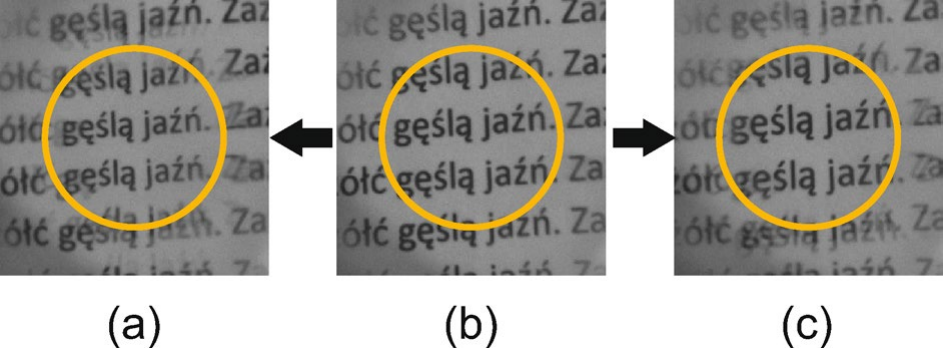
Different images of the lens performance (**a**) $V_1$= 1.4 V$_{\text {RMS}}$ and $V_2 = 4.5$ V$_{\text {RMS}}$ (focused). (**b**) Lens switched off. (**c**) $V_1= 1.85$  V$_{\text {RMS}}$ and $V_2$ = 0.6 V$_{\text {RMS}}$ (focused).

Finally, to demonstrate the image quality generated by this experimental LC mixture, in Fig. 14 different focusing cases are shown. A piece of text is placed in front of the TELCL lens and captured by a camera. Only one polarizer parallel to the alignment direction is used in this case. The yellow circle represents the active area of 1 cm diameter. In Fig. 14b, the voltage is switched off as a starting point. No considerable scattering is observed, even though the thickness of the LC cell in the TELCL is 80 µm. Then, we switch to a negative lens applying the voltages $V_1=1.4$ V$_{\text {RMS}}$ and $V_2 = 4.5$ V$_{\text {RMS}}$, producing an out of focus image that is refocused by adjusting the camera objective (the letters are reduced in size), Fig. 14a). In the same way, when a positive lens is used ($V_1= 1.85$ V$_{\text {RMS}}$ and $V_2 = 0.6$ V$_{\text {RMS}}$), Fig. 14c, the letters increase in size.

## Conclusions

A LC mixture is proposed and experimentally demonstrated to work in a large aperture LC spherical lens. This mixture is made of compounds that are terphenyl and biphenyl derivatives with an isothiocyanate terminal group and fluorinated lateral substituents. The substitution with a strongly polar isothiocyanate group together with an aromatic rigid core provides $\pi$-electron coupling, providing a high birefringence. Specifically, the measured birefringence ranges from 0.49 to 0.318 for a wavelength of 400 nm to 1600 nm. The excellent properties of this LC mixture are demonstrated in a large-aperture LC lens with a maximum optical power of almost − 1.5 Diopters, three times higher than previous reports using the same structure. The high value of birefringence makes this liquid crystal of particular interest not only for lenses but for all kinds of optical phase modulators and optical devices, both in the visible and infrared regions.

## Data Availability

The datasets used and/or analysed during the current study available from the corresponding author on reasonable request.
