# Author Correction: A high birefringence liquid crystal for lenses with large aperture

**DOI:** 10.1038/s41598-022-20631-8

**Published:** 2022-09-29

**Authors:** N. Bennis, T. Jankowski, O. Strzezysz, A. Pakuła, D. C. Zografopoulos, P. Perkowski, J. M. Sánchez-Pena, J. M. López-Higuera, J. F. Algorri

**Affiliations:** 1 Institute of Applied Physics, Military University of Technology, Kaliskiego 2, 00-908 Warsaw, Poland; 2 Faculty of Mechatronics, Warsaw University of Technology, Św. Andrzeja Boboli 8, 02-525 Warsaw, Poland; 3 Institute of Chemistry, Military University of Technology, Kaliskiego 2, 00-908 Warsaw, Poland; 4 Consiglio Nazionale delle Ricerche, Istituto per la Microelettronica e Microsistemi (CNR-IMM), 00133 Rome, Italy; 5 Department of Electronic Technology, Carlos III University, 28911 Madrid, Spain; 6 Photonics Engineering Group, Universidad de Cantabria, 39005 Santander, Spain; 7 CIBER de BioingenieraBiomateriales y Nanomedicina, Instituto de Salud Carlos III, 28029 Madrid, Spain; 8 Instituto de Investigación Sanitaria Valdecilla (IDIVAL), 39011 Santander, Spain

Correction to: *Scientific Reports* https://doi.org/10.1038/s41598-022-18530-z, published online 26 August 2022

The original version of this Article contained an error in the spelling of the author A. Pakuła which was incorrectly given as A.Pakua.

The original Article has been corrected.

